# Draft Genome Sequencing of an Acinetobacter ursingii Isolate from Healthy Human Skin, Carrying Multidrug Resistance Genes

**DOI:** 10.1128/genomeA.00394-18

**Published:** 2018-05-10

**Authors:** Lenka Palkova, Gabriel Minarik, Katarina Soltys

**Affiliations:** a Institute of Molecular Biomedicine, Faculty of Medicine, Comenius University, Bratislava, Slovakia; b Geneton, Ltd., Bratislava, Slovakia; c Comenius University Science Park, Bratislava, Slovakia

## Abstract

In this paper, we report the data from whole-genome shotgun sequencing of an Acinetobacter ursingii isolate from healthy human skin of the forearm. The bacterial genome includes 3,473 genes and carries beta-lactamase resistance genes as well as resistance genes for several heavy metals.

## GENOME ANNOUNCEMENT

Although Acinetobacter spp. are naturally present on human skin, this bacterium can act as a potential pathogen and cause severe bacteremia (1–3). It is capable of long-term survival and transfer in intensive care units. These characteristics, in combination with increasing antibiotic resistance, make this bacterium considerably frightening (4, 5).

Acinetobacter ursingii is a relatively new species (6), and its identification is still problematic (7). This may contribute to fact that among other Acinetobacter spp., A. ursingii is rarely identified (8). Here, we report results of whole-genome shotgun sequencing of an A. ursingii isolate, strain blaTEM-116. The sample was obtained from the collection of bacterial strains of the Department of Molecular Biology, Comenius University, Bratislava, Slovakia. It was defined to be an isolate from healthy human skin obtained from an individual in a nonclinical environment. A single bacterial colony was cultivated on MacConkey agar (Sigma-Aldrich, Munich, Germany). Genomic DNA was isolated using a DNeasy blood and tissue kit (Qiagen, Hilden, Germany). A transposon-based sequencing library was prepared using a Nextera XT kit (Illumina, San Diego, CA). The DNA profile of the library was verified by using an Agilent 2100 Bioanalyzer (Agilent Technologies, Santa Clara, CA, USA) and quantified with a Qubit 2.0 fluorometer (Thermo Fisher Scientific, Waltham, USA). The library was sequenced using paired-end sequencing on an Illumina MiSeq platform (Illumina, San Diego, CA, USA). The quality of reads was checked with the FastQC tool. *De novo* assembly was performed with CLC Genomics Workbench (length fraction, 0.5; similarity fraction, 0.8). Annotation of the sequence was performed with the NCBI Prokaryotic Genome Annotation Pipeline using the best-placed reference set with GeneMarkS+ v. 4.4 (https://www.ncbi.nlm.nih.gov/genome/annotation_prok/).

A total of 3,079,326 paired reads and 636,809 single sequencing reads were obtained and assembled into 194 contigs longer than 200 bp, with 40% GC content. The A. ursingii isolate was identified according to the whole-genome sequencing (WGS) database of GenBank. The total DNA sequence is 3,578,188 bp long and provides 3,473 genes. These include 3,236 coding genes and 78 RNA genes (12 rRNAs, 62 tRNAs, and 4 noncoding RNAs [ncRNAs]). There was no plasmid detected using the online tool PlasmidFinder (https://cge.cbs.dtu.dk/services/PlasmidFinder/).

A remarkable finding was the presence of the following resistance genes for heavy metals: copper (multicopper oxidase, *copB*, *copC*, *copD*, *cutE*, *corC*, *cusR*, and copper-translocating P-type ATPase), tellurium (*terZ*), arsenic (*arsB*, *arsH*, *arsR*, *arsC*, and *acr3*), cobalt-zinc-cadmium complex (*cusA*, *cusB*, *czcA*, *czsB*, *czcD*, *czrR*, and TR), and chromium compounds (*chrA*).

In the bacterium genome, beta-lactamase resistance genes class A (TEM-116) and class C (BL and BLc) were found, as well as the following genes involved in drug resistance machinery: multidrug resistance efflux/transporter genes (Bcr/CflA family, RND, OML, *cmeB*, *cmeC*, MATE family of MDR efflux pumps, *macA*, *macB*, *tolC*, and *arcB*), toxic anion resistance protein, glyoxalase/bleomycin resistance/extradiol dioxygenase family protein, organic hydroperoxide resistance protein, methyl viologen resistance protein smvA, hdeD family acid-resistance protein, chemical-damaging agent resistance protein C, resistance-to-fluoroquinols genes (*gyrA*, and *gyrB*), and bile hydrolysis genes (*bsh*, and AKGTO).

Furthermore, we found a cluster of genes associated with colicin V and bacteriocin production (*dedA*, *dedE*, R1, R3, R4, R5, and *purF*) and other virulence-associated genes and genes associated with invasion and intercellular resistance, including active virulence operons involved in protein synthesis (*rv0682*, *rv0683*, *rv0684*, *rv0685*, *rv1641*, *rv1642*, and *rv1643*), DNA transcription (*rv0667* and *rv0668*), quinolinate biosynthesis (*rv1594*, *rv1595*, and *rv1596*), and internalin-like proteins.

### Accession number(s).

This whole-genome shotgun project has been deposited in GenBank under the accession no. PTPV00000000. The version described in this paper is version PTPV01000000 and consists of sequences PTPV01000001 to PTPV01000194. The sequence of the 16S rRNA gene has been deposited in GenBank under the accession no. MH113156.
